# An Unusual Case of Polymicrobial Bacteremia From Methicillin-Resistant Staphylococcus Aureus and Shigella

**DOI:** 10.7759/cureus.12011

**Published:** 2020-12-10

**Authors:** Elona Shehi, Haider Ghazanfar, Ked Fortuzi, Danial Shaikh, Anil Dev

**Affiliations:** 1 Medicine/Gastroenterology, BronxCare Health System, Bronx, USA; 2 Internal Medicine, BronxCare Health System, Bronx, USA; 3 Internal Medicine, BronxCare Hospital, Bronx, USA; 4 Gastroenterology, BronxCare Health System, Bronx, USA

**Keywords:** polymicrobial bacteremia, shigella, methicillin-resistant staphylococcus aureus, human immunodeficiency virus, morbidity

## Abstract

Bloodstream infections (BSIs) are a significant cause of morbidity and mortality worldwide. Patients with polymicrobial BSI have a two-fold risk of hospital mortality as compared with patients with monomicrobial BSI. We present a case of a 53-year-old African American male with a medical history significant for hyperlipidemia, coronary artery disease, hypertension, anxiety, depression, and human immunodeficiency virus non-adherent to antiretroviral therapy who presented to the hospital with complaints of shoulder pain and diarrhea. The physical exam was significant for multiple skin abscesses, the largest being 5x6 cm. Blood culture grew Shigella and methicillin-resistant Staphylococcus aureus (MRSA), stool culture grew Shigella, and wound culture after incision and drainage grew MRSA. Transthoracic echocardiogram showed no vegetations. He was treated with vancomycin and ceftriaxone. The patient's clinical condition improved, and diarrhea resolved. Patient repeat cultures showed no growth. As polymicrobial bacteremia is associated with higher morbidity and mortality, early initiation of antibiotics and appropriate antibiotic therapy are pivotal.

## Introduction

Bloodstream infections (BSIs) are a major cause of morbidity and mortality worldwide, with an estimated annual national incidence of around half a million cases with and mortality rate of approximately 90,000 a year [1]. Polymicrobial bacteremia is defined as bacteremia due to at least two different organisms isolated from the same blood sample [2].

Shigella is a common cause of gastroenteritis; however, bacteremia secondary to Shigella is a rare occurrence. Children, malnourished individuals, and immunocompromised adults such as those with acquired immunodeficiency syndrome (AIDS) have been described as an at-risk population. While bacteremia due to staphylococcus aureus alone is common, there have been no reports to date of polybacterial bacteremia secondary to methicillin-resistant Staphylococcus aureus (MRSA) and Shigella.

We present a case of a 53-year-old male who presented to our institute with generalized weakness and diarrhea and was diagnosed with Shigella and MRSA bacteremia.

## Case presentation

A 53-year-old African American male presented to our institute with complaints of bilateral upper extremity pain, generalized weakness, and watery diarrhea for two weeks. He described diarrhea as 7 to 8 episodes of watery, non-bloody bowel movements. He denied any abdominal pain, nausea, or vomiting. The patient had a medical history significant for hyperlipidemia, coronary artery disease, hypertension, anxiety, depression, and HIV infection, for which he was not taking antiretroviral therapy. He denied any recent travel, sick contacts, hospitalizations, or any recent antibiotic use. The review of systems was otherwise unremarkable. He was an active smoker, a former intravenous drug user, and occasionally drank alcohol socially. He was sexually active with multiple male partners. Vitals signs showed a blood pressure of 116/80 mmHg, a pulse of 105 beats per minute, and a temperature of 98.1°F. The patient had multiple skin abscesses on inspection: two in the left axillar region (2x3 cm), and one on the left shoulder (6x5 cm). The cardiopulmonary, neurological, and abdominal examinations were within normal limits. The rectal exam revealed a decreased anal sphincter tone and loose brown stools. His initial laboratory results are summarized in Table 1.

**Table 1:** Laboratory investigation on admission

Investigation	Value	Normal Value Range
Complete Blood Cell Count		
White Blood Cell Count	7.7 k/ul	4.8 - 10.8 k/ul
Hemoglobin	10.3 g/dl	12.0 - 16.0 g/dl
Hematocrit	31.80%	42.0% to 51.0%
Platelet	181 k/ul	150 - 400 k/ul
Basic Metabolic Profile		
Sodium	127 mEq/L	135 - 145 mEq/L
Potassium	5.6 mEq/L	3.5 - 5.0 mEq/L
Sodium Bicarbonate	18 mEq/L	24 - 30 mEq/L
Chloride	90 m Eq/L	98- 108 mEq/L
Glucose	132 mg/dL	70 - 120 mg/dL
Blood Urea Nitrogen	58 mg/dL	8 - 26 mg/dL
Creatinine	4.2 mg/dL	0.5 - 1.5 mg/dL
Calcium	8.5 mg/dL	8.5 - 10.5 mg/dL
Urine Studies		
Sodium, Urine	24 mEq/L	40 -220 mEq/L
Creatinine, Urine	221 mg/dL	20 - 200 mg/dL
Potassium, Urine	72 mEq/L	22- 160 mEq/L
Chloride, Urine	< 20 mEq/L	110 - 250 mEq/L

The patient was started on intravenous (IV) antibiotics with ceftriaxone and vancomycin and was admitted to the medical ward for further care. He underwent incision and drainage of the abscesses, cultures of which returned positive for MRSA. The patient's stool was sent for further analysis. Studies were negative for Clostridium difficile, however positive for Shigella. His blood culture also returned positive for Shigella from the anaerobic bottle and MRSA from the aerobic bottle. Shigella was sensitive to ceftriaxone, levofloxacin, and linezolid; while MRSA was susceptible to vancomycin, daptomycin, linezolid, tetracycline, and gentamicin. The stool studies and blood culture results are summarized in Table 2.

**Table 2:** Stool analysis and blood culture results MRSA, methicillin-resistant Staphylococcus aureus

Investigation	Result
pH, Stool	7.6 [7.0 - 7.5 pH units]
Preserved Stool	No ova or parasites are seen
Fecal Leukocyte Stain	Moderate Leukocytes are seen
R/O Cryptosporidium	Not Detected
Stool Culture	Shigella growth
H.Pylori Ag, ql, stool	Not Detected
Clostridium difficile toxin, stool	Not Detected
Giardia Lamblia Antigen Assay	Not Detected
Cyclospora/Isospora,	Not Detected
Ova and parasites	Negative on
Fecal calprotectin	5690 mcg/g
Blood Culture	MRSA and Shigella

An infectious diseases specialist was consulted who recommended switching vancomycin to daptomycin because of worsening renal function and continuing IV antibiotics to complete two therapy weeks. Concerns for hemolytic uremic syndrome were dispelled after renal function recovery with IV fluids and a change of antibiotics. Over the next few days, the patient's clinical condition improved. A repeat set of blood cultures was negative for microorganisms. His diarrhea improved, and the patient was discharged with a course of oral doxycycline for two more weeks. 

## Discussion

The incidence of polymicrobial BSI has increased over the past decade. Patients with polymicrobial BSI have a two-fold risk of in-hospital mortality compared to patients with monomicrobial BSI [3]. Staphylococcus aureus (S. aureus) is a leading cause of community-acquired and healthcare-associated bacteremia. The annual incidence of S. aureus bacteremia (SAB) in the United States is 38.2 to 45.7 per 100,000 person-years [4,5], and in-patient infection rates have been on the rise [6]. Polymicrobial SAB is rare as compared to monomicrobial SAB. According to a study done by Park et al., it was concluded that polymicrobial SAB was also associated with a more severe course of illness [7]. The most common organism isolated alongside polymicrobial SAB is enterococcus spp., and the most significant risk factors for polymicrobial SAB are neutropenia, biliary tract catheters, and intra-abdominal infections [7]. Our patient did not have any of the above-mentioned risk factors, and the most likely source of S. aureus inoculum in our case was the axillary abscesses.

To the best of our knowledge, there are no reports of polymicrobial SAB with Shigella. Shigella species are a common cause of diarrhea worldwide and a significant cause of morbidity and mortality, especially in developing countries. Shigella organisms can survive transit through the stomach since they are less susceptible to acid than other bacteria, and for this reason, as few as 10 to 100 organisms can cause disease [8]. Shigella's transmission occurs via contaminated food or water and via direct person-to-person contact. Shigellosis has been reported in men who have sex with men (MSM), as in our case. In a study of 235 MSM with gastroenteritis, Shigella was detected in 30.5% of diarrheal episodes [9]. Shigella invades the colonic mucosal cells, causing an intense inflammatory response, which leads to epithelial cell death, the formation of mucosal ulceration, and abscesses. Although Shigella infection is limited to the intestinal mucosa, rarely bacteremia due to Shigella can occur, especially in immunocompromised individuals. In a study of systemic Shigellosis from South Africa, HIV-infected patients were substantially more likely to die than HIV-uninfected individuals [10]. Thus, timely evaluation and initiation of antibiotics in concordance with an infectious diseases expert, such as in our case, is necessary for these individuals.

## Conclusions

Our case highlights the occurrence of two rare entities, bacteremia secondary to Shigella and polymicrobial BSI, unfortunately manifesting together as one. A serious health hazard on their own, jointly polymicrobial bacteremia secondary to Shigella and MRSA, only serve to increase the risk of morbidity and mortality. Our case had identifiable risk factors of HIV infection and MSM for Shigella bacteremia; whereas the axillary abscesses were the SAB source. Early consultation with an infectious diseases expert and appropriate antibiotic therapy initiation is paramount to improve survival in such cases.
